# A New Leptoceratopsid (Ornithischia: Ceratopsia) from the Upper Cretaceous of Shandong, China and Its Implications for Neoceratopsian Evolution

**DOI:** 10.1371/journal.pone.0013835

**Published:** 2010-11-04

**Authors:** Xing Xu, Kebai Wang, Xijin Zhao, Corwin Sullivan, Shuqing Chen

**Affiliations:** 1 Key Laboratory of Evolutionary Systematics of Vertebrates, Institute of Vertebrate Paleontology and Paleoanthropology, Chinese Academy of Sciences, Beijing, China; 2 Zhucheng Dinosaur Museum, Bureau of Tourism, Zhucheng, Shandong, China; Raymond M. Alf Museum of Paleontology, United States of America

## Abstract

**Background:**

The ceratopsians represent one of the last dinosaurian radiations. Traditionally the only universally accepted speciose clade within the group was the Ceratopsidae. However, recent discoveries and phylogenetic analyses have led to the recognition of a new speciose clade, the Leptoceratopsidae, which is predominantly known from the Upper Cretaceous of North America.

**Methodology/Principal Findings:**

Here we report a new leptoceratopsid taxon, *Zhuchengceratops inexpectus* gen. et sp. nov., based on a partial, articulated skeleton recovered from the Upper Cretaceous Wangshi Group of Zhucheng, Shandong Province, China. Although *Zhuchengceratops* is significantly different from other known leptoceratopsids, it is recovered as a derived member of the group by our phylogenetic analysis. Furthermore, *Zhuchengceratops* exhibits several features previously unknown in leptoceratopsids but seen in ceratopsids and their close relatives, suggesting that the distribution of morphological features within ceratopsians is more complex than previously realized.

**Conclusion/Significance:**

The discovery of *Zhuchengceratops* increases both the taxonomic diversity and the morphological disparity of the Leptoceratopsidae, providing further support for the hypothesis that this clade represents a successful radiation of horned dinosaurs in parallel with the Ceratopsidae in the Late Cretaceous. This documents a surprising case of the coexistence and radiation of two closely-related lineages with contrasting suites of jaw and dental features that probably reflect adaptation to different food resources.

## Introduction

The leptoceratopsids are a group of small, quadrupedal horned dinosaurs. They normally have a total body length of about two meters, and are thus much smaller than the contemporary ceratopsids [1]. The leptoceratopsids are characterized by robust jaws equipped with highly specialized large teeth and, unlike derived horned dinosaurs, lack horns and bear an extremely short frill [1], [2]. The Leptoceratopsidae was named implicitly by Nopcsa in 1923 [3], and explicitly by Makovicky in 2001 [4]. Leptoceratopsids have only recently been recognized as a distinctive group of ceratopsian dinosaurs [2], [4]. Known fossil occurrences of leptoceratopsids are mainly limited to the Upper Cretaceous of North America [5], though two taxa have been previously described from the Upper Cretaceous of Asia [4], [6]–[8]. Other reported leptoceratopsid occurrences include some dental material from the Upper Cretaceous of Europe [5] and one controversial taxon from the Lower Cretaceous of Australia [9]. Although leptoceratopsids are considered by some studies [2], [4] to be a relatively basal clade within the Neoceratopsia, some members of the group coexisted with derived ceratopsids in the latest Cretaceous [10].

In the summer of 2008, we excavated the bone-beds of the Upper Cretaceous Wangshi Group at the Kugou locality, Zhucheng, Shandong Province. The site is extremely rich in disarticulated bones of *Shantungosaurus*, the largest known hadrosaurid [11], [12]. To our surprise, a partial, articulated medium-sized ceratopsian skeleton (Table 1) was also recovered from the bone-beds. It possesses morphological features that identify it as a member of a new leptoceratopsid taxon. In the present paper, we describe the new specimen from the Kugou locality and discuss its implications for understanding the evolution of the leptoceratopsid dinosaurs.

**Table 1:** Selected measurements (in cm) of ZCDM V0015.

Mandible length	47.0
Predentary length	25.0*
Dentary length	35.0
Dentary anterior end height	24.0
Dentary minimum height	13.5
Dentary coronoid region height	19.0
Axial centrum length	4.5
Axial centrum height	4.0
3^rd^ cervical centrum length	3.5
3^rd^ cervical centrum height	4.5
4^th^ cervical centrum length	2.5
4^th^ cervical centrum height	5.0
5^th^ cervical centrum length	3.5
5^th^ cervical centrum height	5.0
6^th^ cervical centrum length	3.5
6^th^ cervical centrum height	5.0
7^th^ cervical centrum length	3.5
7^th^ cervical centrum height	4.0
8^th^ cervical centrum length	4.0
8^th^ cervical centrum height	4.0
9^th^ cervical centrum length	3.5
9^th^ cervical centrum height	4.0
10^th^ cervical centrum length	3.5
10^th^ cervical centrum height	5.0
1^st^ dorsal centrum length	3.5
1^st^ dorsal centrum centrum height	5.5
2^nd^ dorsal centrum length	3.5
2^nd^ dorsal centrum centrum height	6.0
Atlantal rib length	6.0*
Axial rib length	12.0#

*estimated length.

# incomplete measurement.

all centrum heights were measured at the posterior end.

## Methods

### Fossil Preparation

The holotype specimen was prepared free of matrix, and most of the individual bones were separated (Figures 1,2,3,4,5,6,7,8). However, a cast was made before the specimen was dismantled (Figure 1A), in order to record the relative positions of the elements as they were preserved.

**Figure 1:**
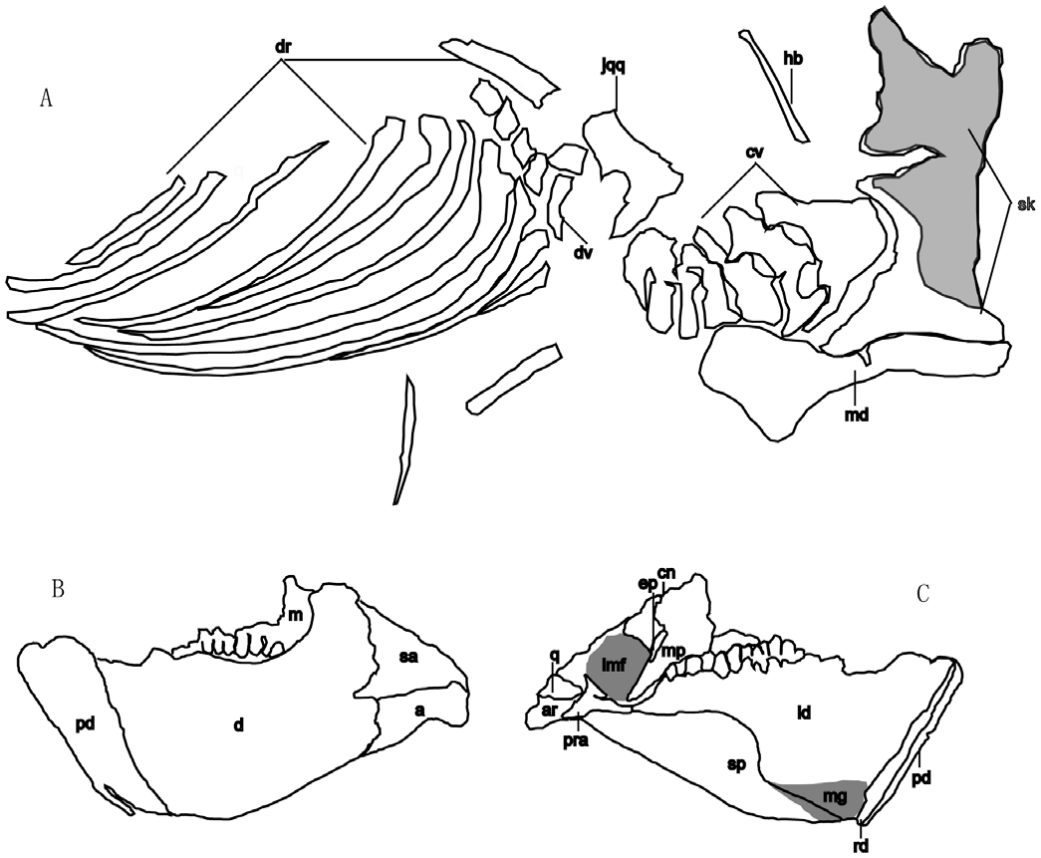
*Zhuchengceratops inexpectus* holotype (ZCDM V0015). Line-drawings of *Zhuchengceratops inexpectus* holotype as preserved (A), and of left side of skull and mandible in lateral (B) and medial (C) views. The gray area in (A) represents the impression on the matrix made by the right maxilla. Abbreviations: a, angular; ar, articular; cn, coronoid; cv, cervical vertebrae; dv, dorsal vertebrae; ep, ectopterygoid; hb, hyoid bone; imf, internal mandibular fossa; jqq, jugal-quadratojugal-quadrate;ld, left dentary; m, maxilla; md, mandible; mg, Meckelian groove; mp, mandibular process; pd, predentary; pra, prearticular; q, quadrate; rd, right dentary; sa, surangular; sk, skull; sp, splenial.

**Figure 2:**
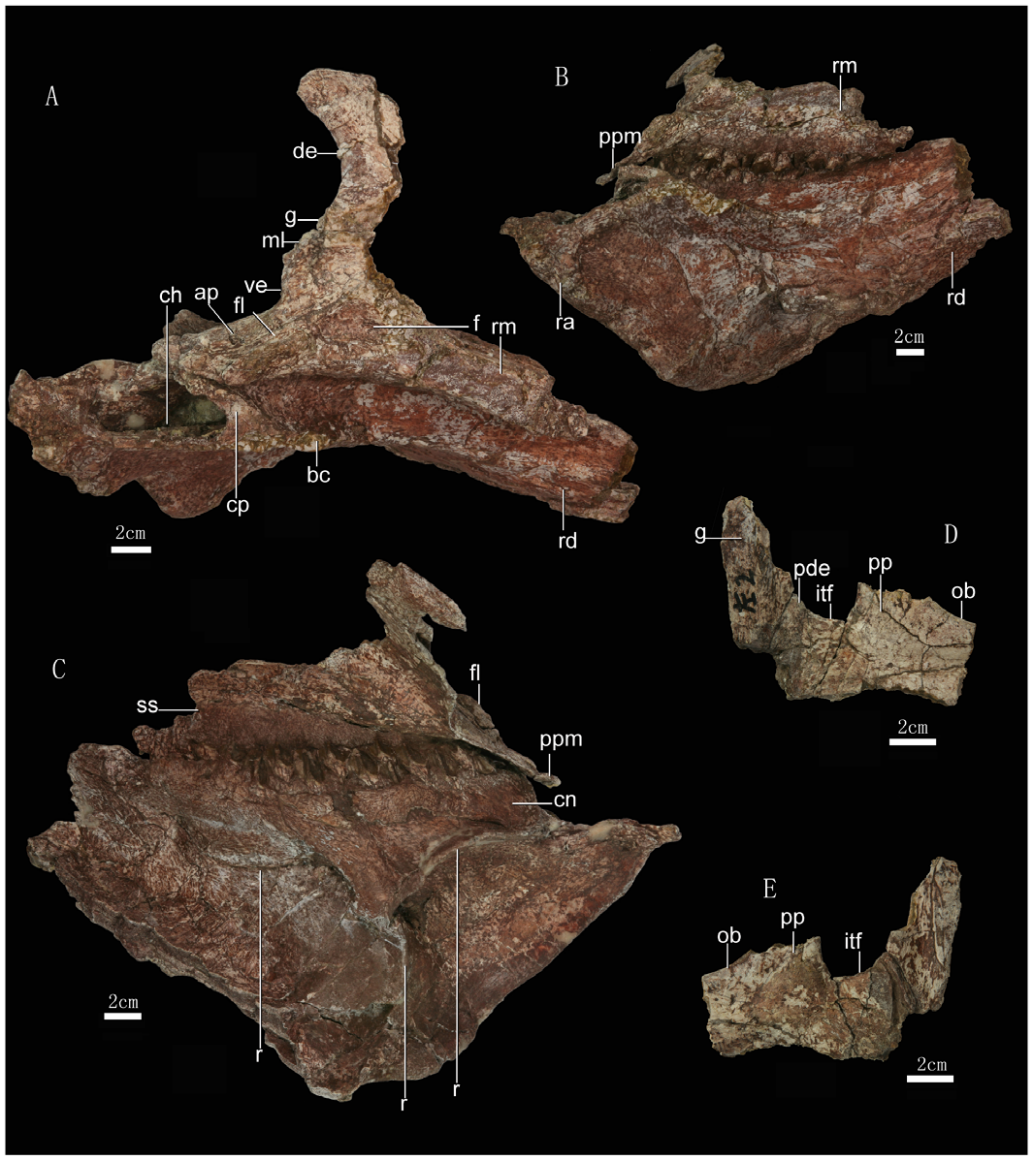
Right maxilla, jugal, quadratojugal, ectopterygoid, and mandible of *Zhuchengceratops inexpectus* holotype (ZCDM V0015). Right maxilla and mandible in dorsolateral (A), lateral (B), and medial (C) views; right jugal, quadratojugal, and quadrate in lateral (D) and medial (E) views. Abbreviations: ap, articular facet for pterygoid; bc, buccal crest; ch, chamber within coronoid process; cn, coronoid; cp, coronoid process; de, dorsal embayment; f, fossa; fl, flange; g, groove; itf, infratemporal fenestra; ml, medial lamina; ob, orbit; pde, posterodorsal expansion; pp, postorbital process; ppm, posterior process on maxilla; r, ridge; ra, right angular; rd, right dentary; rm, right maxilla; ss, strap-like surface; ve, ventral embayment.

**Figure 3:**
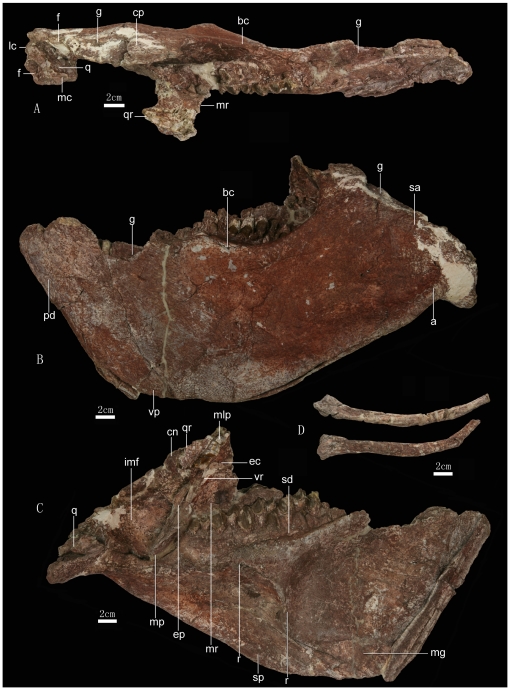
Left maxilla, pterygoid, ectopterygoid, and mandible of *Zhuchengceratops inexpectus* holotype (ZCDM V0015). Left maxilla, pterygoid, ectopterygoid, and mandible in dorsal (A), lateral (B), and medial (C) views; D, a pair of hyoid bones. Abbreviations: a, angular; bc, buccal crest; cn, coronoid; cp, coronoid process; ec, “Eustachian canal”; ep, ectopterygoid; f, fossa; g, groove; imf, internal mandibular fossa; lc, lateral condyle; mc, medial condyle; mg, Meckelian groove; mlp, mid-line process; mp, mandibular process; mr, maxillary ramus; pd, predentary; q, quadrate; qr, quadrate ramus; r, ridge; sa, surangular; sd, supradentary; sp, splenial; vp, ventral process; vr, ventral ridge.

**Figure 4:**
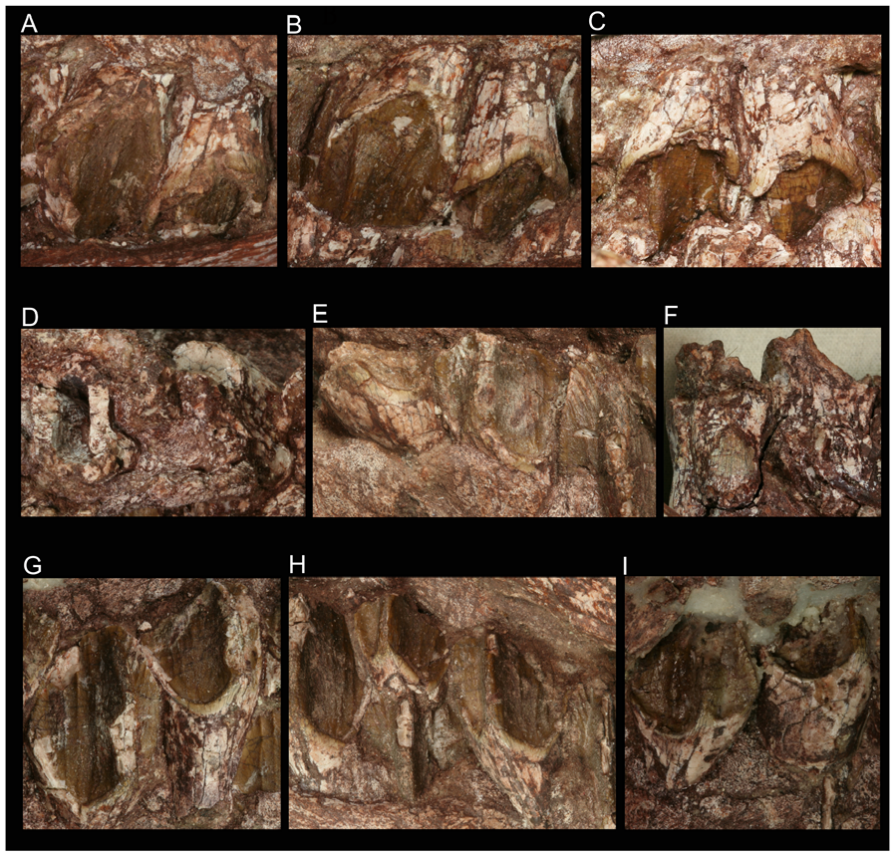
Dentition of *Zhuchengceratops inexpectus* holotype (ZCDM V0015). Two anterior-middle (A), two middle (B), and two posterior (C) right maxillary teeth in labial view; D, two middle left maxillary tooth roots in cross-section; E, three anterior right dentary teeth in lingual view; F, two anterior left dentary teeth in labial view; G, two middle right dentary teeth in lingual view; H, three middle-posterior right dentary teeth in lingual view; I, two posterior left dentary teeth in lingual view. Not to scale.

**Figure 5:**
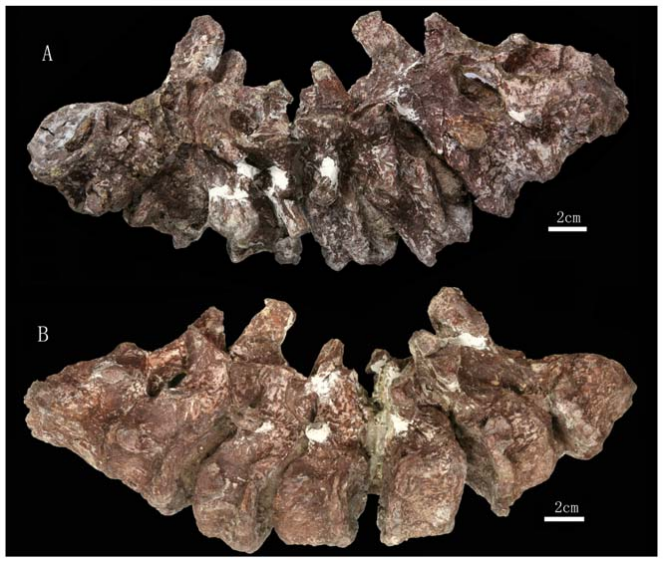
The 1^st^ through 8^th^ presacral vertebrae preserved in the *Zhuchengceratops inexpectus* holotype (ZCDM V0015). The 1^st^ through 8^th^ presacral vertebrae in right lateral (A) and left lateral (B) views.

**Figure 6:**
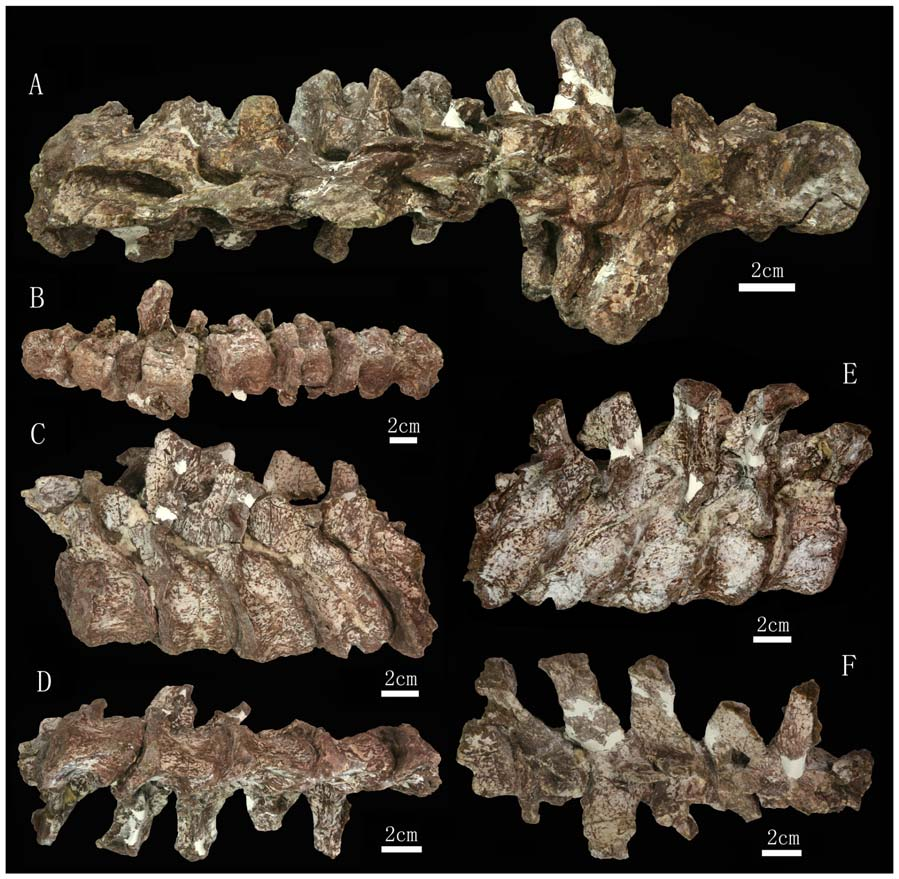
The 1^th^ through 13^th^ presacral vertebrae preserved in the *Zhuchengceratops inexpectus* holotype (ZCDM V0015). The 1^st^ through 8^th^ presacral vertebrae in dorsal (A) and ventral (B) views; the 9^th^ through 13^th^ presacral vertebrae in left lateral (C), right lateral (D), ventral (E), and dorsal (F) views.

**Figure 7:**
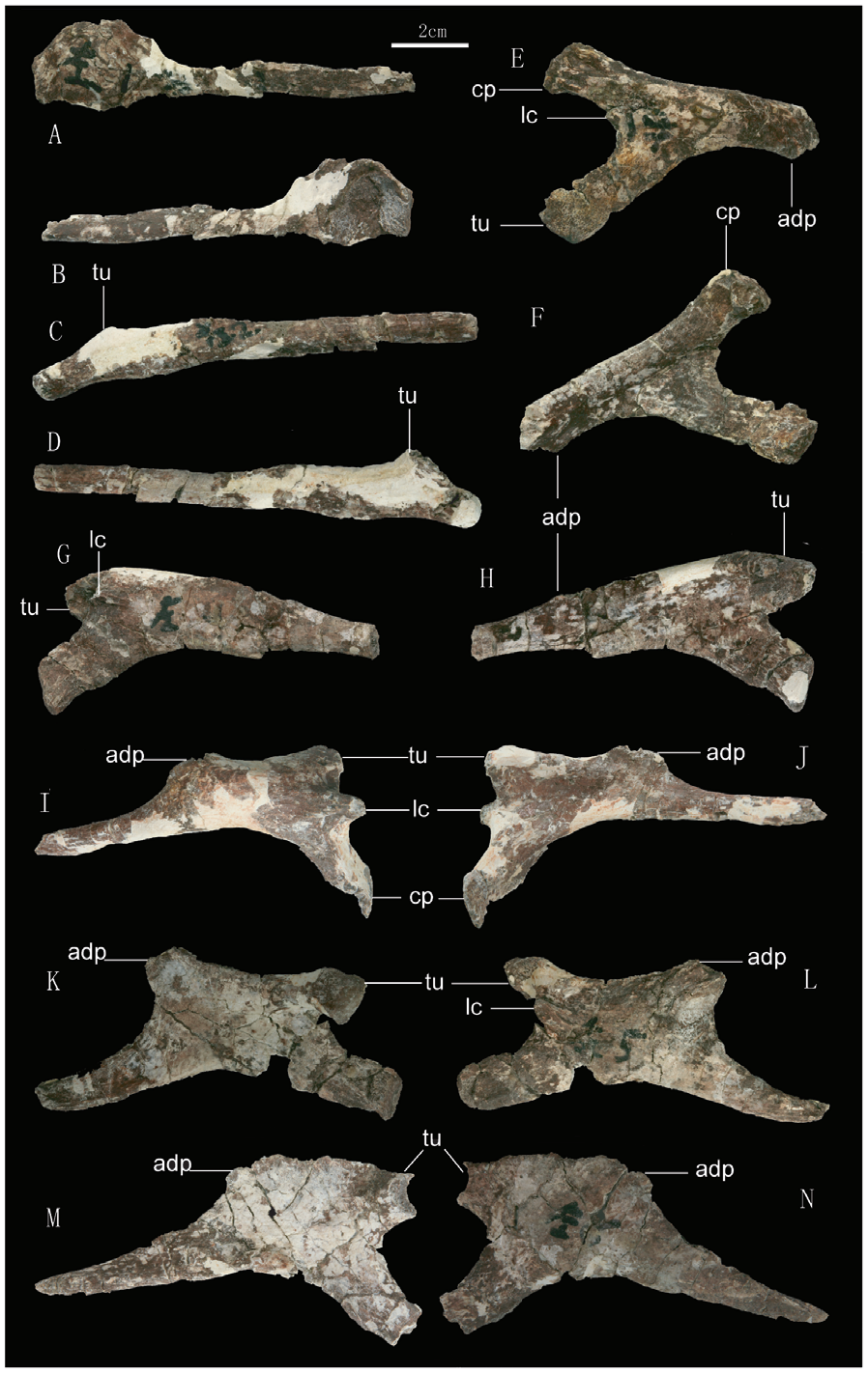
Preserved cervical ribs of *Zhuchengceratops inexpectus* holotype (ZCDM V0015). Atlantal rib in lateral (A) and medial (B) views; axial rib in lateral (C) and medial (D) views; 3^rd^ cervical rib in lateral (E) and medial (F) views; 4^th^ cervical rib in lateral (G) and medial (H) views; 5^th^ cervical rib in lateral (I) and medial (J) views; 6^th^ cervical rib in medial (K) and lateral (L) views; and a posterior cervical rib in lateral (M) and medial (N) views. Abbreviations: adp, accessory dorsal process; cp, capitulum; lc, lateral crest; tu, tuberculum.

**Figure 8:**
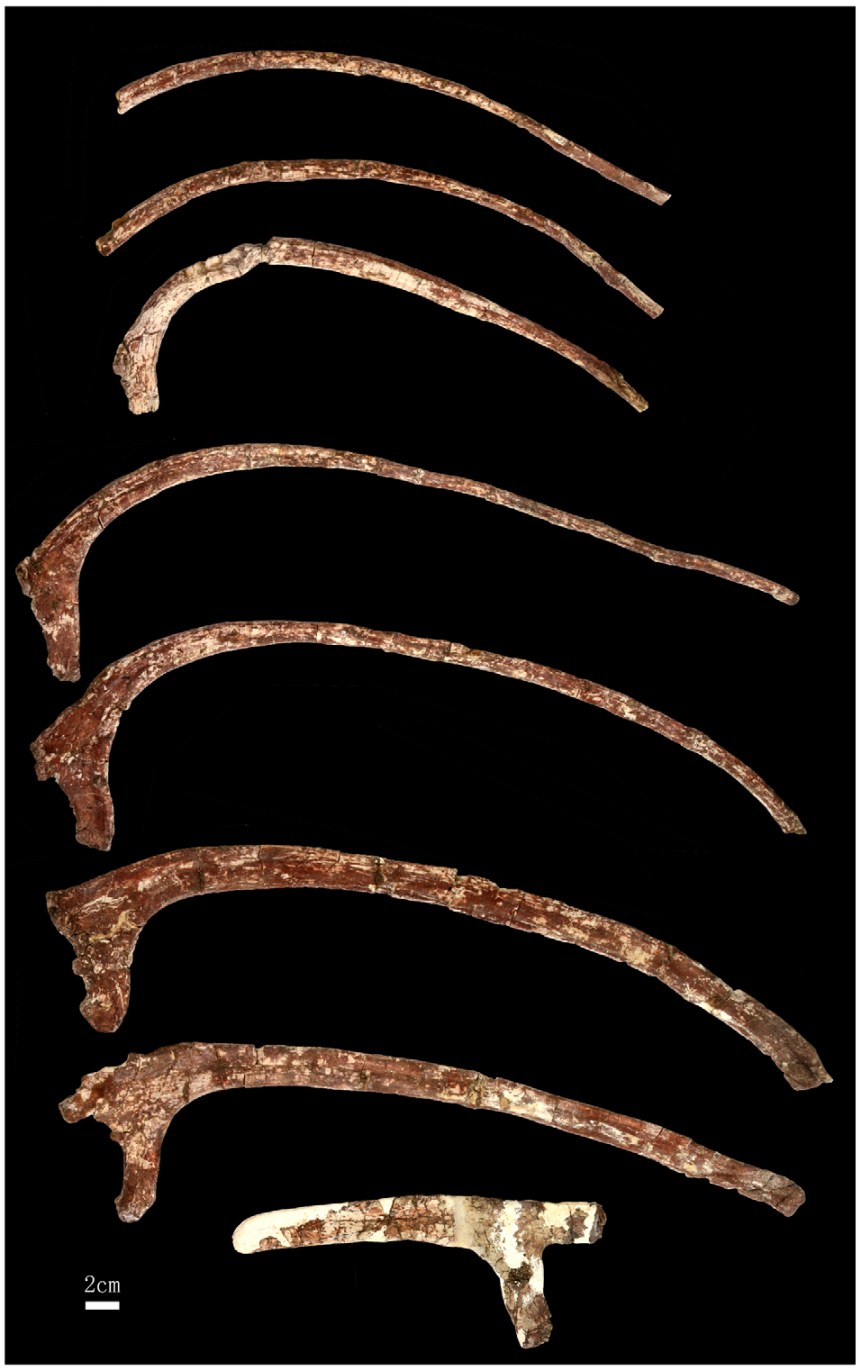
Preserved dorsal ribs of *Zhuchengceratops inexpectus* holotype (ZCDM V0015). Anterior dorsal ribs at bottom and posterior dorsal ribs at top.

### Terminology

The terms ‘anterior’ and ‘posterior’ are used with respect to both the placement of osteological elements (e.g. anterior and posterior dorsal vertebrae) and different portions of each individual element (the anterior and posterior ends of the mandible). We use previously established terminology for vertebral laminae [13].

### Phylogenetic Analysis

**Figure 9:**
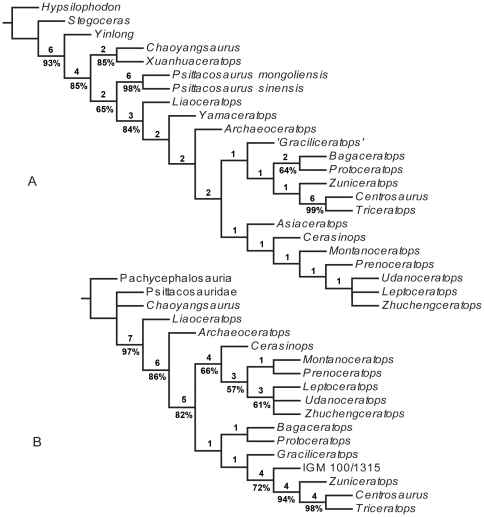
Suggested systematic position of *Zhuchengceratops inexpectus* among the Ceratopsia. A, the strict consensus of three most parsimonious trees produced by our analysis of Makovicky's (2010) matrix with *Zhuchengceratops inexpectus* added in; B, the strict consensus of six most parsimonious trees produced by our analysis of Chinnery and Horner's (2007) matrix with *Zhuchengceratops inexpectus* added in. Bremer support and bootstrap values are labeled (bootstrap values below 50% are not shown).

In order to assess the systematic position of the taxon described in this paper, we coded it separately (Table 2) into two substantially different data matrices recently published for ceratopsians [7], [14]. The two matrices were both analyzed using the software package TNT [15], with the implicit enumeration search strategy. All parameters were left at their default settings, with the following exceptions: maximum trees in memory  = 10000, and collapsing rules = 0 max. length. One analysis resulted in three most parsimonious trees (tree length = 280 steps, CI = 0.63, and RI = 0.77), shown in Figure 9A. The other analysis resulted in six most parsimonious trees (tree length = 238 steps, CI = 0.61, and RI = 0.71), the strict consensus of which is shown in Figure 9B. We also used TNT to run Bremer Support and Bootstrap analysis (1000 replicates) on the two sets of datasets. The results are indicated in Figures 9A and B.

**Table 2:** Scorings for *Zhuchengceratops inexpectus*.

Makovicky matrix	1????????????????????1???1?????????????????1111111??????????????????10??1100001011100001?101???02211011011????010?????????????????????0???0?????101
Chinnery and Horner matrix	?????????1?????11???????????????????????????????1???????111?11?111100??0022011101?1??????????????????????10010???0001?????

## Results

### Systematic paleontology

Ornithischia Seeley, 1888 [16]

Ceratopsia Marsh, 1890 [17]

Leptoceratopsidae Nopcsa, 1923 [3]

*Zhuchengceratops inexpectus* gen. et sp. nov.

#### Etymology

Genus name from *Zhucheng* (the county that includes the type locality) and *ceratops* (horn-face, Latinized Greek); species name *inexpectus* refers to the unexpected discovery of an articulated skeleton in the Zhucheng bone-beds.

#### Holotype

Zhucheng Dinosaur Museum (ZCDM) V0015, a partial, articulated skeleton including partial maxillae, partial right quadratojugal, jugal, and quadrate, partial left ectopterygoid and pterygoid, nearly complete left and right mandibles, anterior 14 presacral vertebrae and associated ribs, and some additional fragments.

#### Type locality and horizon

Kugou, Zhucheng County, Shandong Province, China. Upper Cretaceous Wangshi Group [11].

#### Diagnosis

A leptoceratopsid with the following autapomorphies: maxilla with a posteroventrally oriented edentulous posterior process, mandible extremely deep (mid-length height about one-third of mandibular length), mandible very thin transversely (ratio of ventral margin width to mandibular length about 0.03), medial surface of mandible with two large fossae separated by thick curved ridge along dentary-splenial contact, predentary sub-vertically oriented (posterior edge forms angle of about 75 degrees with tooth row in lateral view), anterior end of dentary deeper than coronoid region, anterior portion of Meckelian groove floored by both dentary and splenial and much wider than posterior portion, surangular with additional fossa immediately anterior to glenoid fossa, surangular with sharp anteroventral process inserting into dentary in lateral view, articular with semilunate fossa immediately posterior to glenoid fossa, dorsal edge of splenial step-wise in medial view, and middle cervical ribs bifurcated due to presence of prominent accessory dorsal process (condition poorly known in other ceratopsians).

### Description and comparisons

The holotype specimen probably represents an adult individual, judging by the complete closure of the neurocentral sutures of all preserved presacral vertebrae. With a mandibular length of about 50 cm, the *Zhuchengceratops inexpectus* holotype is slightly larger than most adults of *Leptoceratops* [18], [19]. *Zhuchengceratops inexpectus* probably has a very large head as in other neoceratopsians [20], [21], as inferred by comparing the 50-cm-long mandible to the estimated presacral length of about 140 cm.

The right and left maxillae are both incomplete, although the more complete right element preserves most of the ventral and posterior portions (Figures 1,2,3). However, the right maxilla is also represented by a smooth impression on the matrix (Figure 1A), so that the rough shape of the entire element is known. The impression shows that the maxilla was extremely deep dorsoventrally, and even the preserved dorsoventral depth of the right maxilla is more than 150% of the length of the maxillary tooth row (Figures 1A and 2A). A dorsoventrally deep maxilla also appears to be present in *Udanoceratops* [1], [8]. In *Zhuchengceratops*, the alveolar margin is convex in lateral view as in *Leptoceratops* and *Udanoceratops* [22]. Posterior to the alveolar margin is a distinct, posteroventrally directed edentulous process (Figures 2A and B). The length of this posterior process is more than 50 mm, about one-fourth the length of the tooth row. The process is splint-like, dorsoventrally shallow (about 5 mm deep) and mediolaterally wide (about 35 mm). Its posterior end is asymmetric, tapering to a point that lies near the medial edge. The dorsal surface of the process is rough, and probably articulated with the pterygoid and ectopterygoid as in ceratopsids [23]. The dorsolateral edge of this process bears a sub-semicircular flange (Figures 2A and C), and a similar flange appears to be present at the same position on the maxillary posterior process in some ceratopsids [23]. Dorsal to the posterior process the maxilla is strongly deflected medially, but this could be a preservational artifact. There are two embayments along the posterior margin of the maxilla in lateral view, the dorsal embayment being about three times the depth of the ventral one. The ventral part of the dorsal embayment is partially floored by a medial lamina, which forms the medial wall of a groove along the posterior margin of the maxilla within the embayment (Figure 2A). Anterodorsal to the posterior process, the lateral surface of the maxilla bears a distinctive, sub-triangular fossa, posterodorsal to which is a large depression that emanates from the small ventral embayment. In medial view, the ventral part of the maxilla is divided in two by a line of inflection as in some ceratopsids [23]. The dorsal portion faces mediodorsally, whereas the ventral portion is a medially-facing strap-like surface that is deeper anteriorly than posteriorly and covers the maxillary tooth roots. This strap-like surface has a texture different from that of the neighboring bone, and appears to represent a separate skeletal element (Figure 2C). The maxilla probably has a narrow contact with the jugal along its posterodorsal margin, as in ceratopsids [24].

The right jugal and right quadratojugal are incompletely preserved, and we describe the two bones together because the suture between them is not clear. The gently curved orbital margin of the jugal indicates a proportionally large orbit (Figures 1A, 2D and E). The lateral surface of the sub-orbital ramus is concave. The postorbital process is relatively narrow in lateral view compared to the orbit, but wide compared to the infratemporal fenestra: the base of the postorbital process has an anteroposterior width of about 3 cm, similar to the width of the infratemporal fenestra near its ventral border. The jugal appears to expand dorsally at the posterior end to form the posteroventral border of the infratemporal fenestra (Figure 2D), a condition similar to that seen in centrosaurines and some chasmosaurines [24], but this part of the fenestral border may actually be formed by the quadratojugal.

The quadrate is represented by the ventral end of the left element and the middle portion of the right. The preserved portion of the quadrate shaft is sub-triangular in cross section. The medial and anterolateral surfaces of the quadrate are concave whereas the posterolateral surface is nearly flat, though marked by a longitudinal groove (Figure 2D). The ventral end of the quadrate is very robust (about 65 mm wide transversely). The medial condyle of the ventral end of the quadrate protrudes prominently from the shaft.

The pterygoid is represented only by the ventral portion of the left element (Figures 3A–C). However, it is clear that the pterygoid contacts the maxilla more anteriorly than is the case in most non-ceratopsid ceratopsians, over an area including the dorsal surface of the posterior process of the maxilla as well as the medial surface of the bone near the posterior end of the tooth row [10], [25]. The mandibular process of the pterygoid is long, extending ventrally nearly to the ventral border of the internal mandibular fossa (Figure 3C). The mandibular process is in general similar to those of more basal ceratopsians in having a sub-triangular cross section, but the prominent maxillary ramus has a significant ventral extension in the form of a sharp ridge which extends ventrally to the extremity of the mandibular process to border medially a longitudinal groove along the anterior surface of the mandibular process (Figure 3C) as in ceratopsids, *Leptoceratops*, and *Protoceratops* [22]. The maxillary ramus is somewhat twisted, with the dorsal portion displaced laterally relative to the ventral portion. A prominent ventral ridge is present as in ceratopsids and *Protoceratops* [22]. This structure is thin and short, defining a “Eustachian canal”. A large midline process for the opposite pterygoid is present medial to the “Eustachian canal”, but this process is broken (Figure 3C). Lateral to the quadrate ramus the pterygoid forms a short ridge, which combines with the ectopterygoid to define a shallow groove along the posterior surface of the mandibular process. Anterior to this ridge is a distinct fossa. The quadrate ramus is broken away, apart from its relatively narrow base (Figures 3B and C). The quadrate ramus forms a large angle with the maxillary ramus (Figures 3B and C), in contrast to the sharp angle seen in more basal ceratopsians [21], [26].

The ectopterygoid is a small element, about half the length of the mandibular process of the pterygoid. The ectopterygoid is attached to the posterolateral edge of the proximal portion of the mandibular process (Figure 3C). It also contacts the dorsolateral margin of the posterior process of the maxilla, but probably lacks a contact with the jugal. The ectopterygoid is an elongate element, and its orientation is more dorsoventral than anteroposterior. The posterior edge of the ectopterygoid is distinctly curved in lateral view. The medial surface of the ectopterygoid is concave, forming the ventral portion of the groove whose dorsal portion lies on the posterior surface of the mandibular process.

The mandible is massive (Figures 2 and 3). The height at the midpoint (180 mm) is about one-third of the total length, a greater ratio than is known in any other ceratopsian [10], [24]. A massive mandible appears to characterize all known leptoceratopsids [10]. However, the mandible is also very thin transversely, particularly along the ventral margin. The widths of the ventral and dorsal margins are about 15 mm and 50 mm, respectively. The anterior end of the mandible is considerably deeper than the coronoid region, a feature previously unknown in any ceratopsian [1], [10], [24], and the anterior margin is also more vertically oriented in lateral view than in other ceratopsians. However, the lateral profile of the mandible is similar to that of *Leptoceratops* [18] in that the anterior end of the ventral margin of the mandible curves dorsally, whereas the posterior end bends sharply in the ventral direction. Most of the lateral surface of the mandible is concave, especially just anterior to the glenoid region. The long axis of the glenoid fossa is set at about 45 degrees to that of the mandibular ramus. Accordingly, the two rami are inferred to have strongly diverged laterally in the intact mandibular arch, forming an angle of about 90 degrees under the assumption that the long axes of the two glenoid fossae were aligned mediolaterally. The Meckelian groove is anteriorly wide but tapers posteriorly. The anterior portion of the Meckelian groove is floored by both the dentary and the splenial, and the posterior portion by the dentary alone (Figure 3C).

The predentary is more vertically oriented than in other ceratopsians (Figure 3B). The main body of the predentary is sub-rectangular in lateral view, being much shorter anteroposteriorly than high dorsoventrally. The sub-parallel straight anterior and posterior margins each form a large angle with the tooth row (about 75 degrees). In most other ceratopsians, the main body of the predentary tapers to a ventral point in lateral view, with the anterior margin forming a much sharper angle with the tooth row [10]. However, *Protoceratops* is similar to *Zhuchengceratops* in that the ventral portion of the predentary is relatively wide in lateral view. The lateral and ventral processes of the predentary are both small. The ventral process diverges more abruptly from the main body of the predentary than in other ceratopsians, and extends only a very short distance posteriorly.

The large dentary makes a proportionally greater contribution to the lateral surface of the mandible than in other ceratopsians, accounting for more than 60% of the mandibular length. Unlike in other ceratopsians, except *Leptoceratops* [18], the anterior portion of the dentary is strongly expanded dorsoventrally and is the deepest part of the whole dentary. The dorsal margin of the dentary is strongly concave in lateral view. The anterior portion of the margin is considerably dorsal to the dentary tooth row, as is also true to varying degree in other leptoceratopsids. The anterior part of the margin also bears a distinct groove, posteriorly very deep and wide, for reception of the lateral process of the predentary. A broad dentary shelf (more than 30 mm wide) lies lateral to the dentary tooth row (Figure 3B). As in *Bagaceratops* [2], a prominent buccal crest along the lateral edge of the dentary shelf hides nearly half of the tooth row from lateral view. Posteriorly, the lateral surface of the crest is continuous with that of the coronoid process, the distal end of which is slightly expanded anteroposteriorly in a morphology somewhat similar to the derived condition seen in ceratopsids [24]. The laterally situated dentary portion of the coronoid process is slightly constricted at the base in lateral view, and dorsal to this constriction is a distinct bump close to the posterior margin of the process. The posterior end of the dentary tooth row is only slightly anterior to the posterior margin of the coronoid process, a feature more reminiscent of ceratopsids than of other non-ceratopsid ceratopsians. Most of the ventral margin of the dentary is nearly straight, although the anterior portion is slightly convex and the posteriormost part of the margin also slants somewhat dorsally. A few mental foramina are scattered on the lateral surface of the dentary. Posteriorly the dentary has a nearly vertical contact with the surangular and angular, though the former bone sends a pointed anteroventral process into a notch at the posterior margin of the dentary (Figure 3B). A nearly vertical contact with the post-dentary bones is also seen in *Leptoceratops* and ceratopsids, and *Zhuchengceratops* further resembles these taxa in that the contact between the dentary and post-dentary elements lies posterior to the coronoid process. Most of the exposed medial surface of the dentary is occupied by a large, shallow, anteriorly positioned fossa, immediately posterior to which lies a second fossa of much smaller size on the dentary. A sinuous, thick ridge on the dentary marks the line of contact with the splenial and with the posterior edge of the intercoronoid. This ridge defines the dorsal border of the second fossa (Figure 3C). The dentary bears a shelf, albeit a very narrow one, medial to the tooth row.

The post-dentary elements are highly abbreviated, accounting for less than 30% of the total mandibular length. Anteriorly the surangular abuts the ventral portion of the posterior margin of the coronoid process, contacting the dentary laterally and the coronoid medially. The transversely wide dorsal margin of the surangular overhangs the internal mandibular fossa and bears a longitudinal groove (Figure 3B). More posteriorly, the dorsal margin of the surangular widens even further and bears a fossa situated immediately anterior to the glenoid fossa. The edge of the surangular situated lateral to the glenoid fossa does not form a bounding ridge, and in fact is slightly depressed relative to the rest of the dorsal surface of the surangular. The suture between the surangular and dentary is sinuous, but nearly vertically oriented. In most other ceratopsians, an equivalent projection of the surangular is absent. The medial surface of the surangular underlaps the coronoid anterodorsally and forms the floor of the internal mandibular fossa.

The angular is a relatively small element. The anterodorsal portion of the lateral surface is depressed. A long anteroventral process intrudes between the dentary and splenial in lateral view. The posterior end of the angular turns abruptly ventromedially to buttress the articular.

The articular forms the bulk of the glenoid region, which is strongly deflected medially relative to the rest of the mandibular ramus. As a result, the medial edge of the glenoid fossa lies considerably medial to the prearticular. Immediately posterior to the glenoid fossa is a deep semilunate fossa on the dorsal surface of the relatively short retroarticular process (Figure 3A).

The prearticular is a relatively slender element. Anteriorly it contacts the dentary, a derived feature also seen in *Protoceratops, Bagaceratops*, and ceratopsids [2]. A tuberosity is present near the mid-length of the dorsal margin.

The splenial is a large element on the medial surface of the mandible, though it has a small lateral exposure. It forms an anterior process that extends to the level of the mandibular symphysis. The dorsal margin of the anterior process has a somewhat step-wise contact with that of the main body of the splenial. Posteriorly the splenial is pointed, and inserts between the angular and prearticular. Most of the surface of the splenial is depressed, forming a fossa that is dorsally bordered by the sinuous ridge along the dentary-splenial contact (Figure 3C).

The coronoid bone forms the medial surface of the coronoid process (Figure 3C). The dorsal end of the coronoid is strongly expanded anteroposteriorly, matching the dentary portion of the coronoid process, but the ventral part of the bone is much narrower. An elongate, sub-triangular intercoronoid bone (supradentary) is present. The anterior end of this bone is pointed and lies at about the level of the midpoint of the tooth row. The posterior end is deep dorsoventrally and probably contacts the coronoid bone posterior to the dentary tooth row.

Two slender hyoid bones are preserved in association with the mandible (Figure 3D). They are curved and rod-like, each having a wide and flattened posterior end. Rod-shaped hyoid bones are also known in *Leptoceratops* [18].

There are at least 15 maxillary teeth and 15 dentary teeth (Figure 4). At least one dentary alveolus contains two functional teeth (Figure 4H), but most positions have only a single functional tooth. All the teeth are closely packed, and the maxillary and dentary teeth must have sheared past one another almost vertically as in other leptoceratopsids and ceratopsids.

The maxillary teeth are mostly visible only in labial view, since the upper and lower jaws are preserved in a position of close occlusion on either side of the skull. Enamel is present on the labial sides of the maxillary teeth, but absent on the lingual sides. The prominent primary ridge on the labial side of each maxillary tooth is strongly offset, lying at the midpoint of the posterior half of the tooth crown. The primary ridge is considerably lower and wider basally than apically, and is inset from the crown base. At the base, a continuous anteroposterior ridge curves apically at both ends to form prominent anterior and posterior marginal ridges. However, in one of the anterior maxillary teeth this anteroposterior ridge is interrupted by a groove extending onto the labial face of the maxillary tooth root. Two or three extremely weak secondary ridges are present anterior to the primary ridge on the labial surface of most, but not all, maxillary tooth crowns. Little can be said about the lingual faces of the maxillary teeth. However, a large, nearly vertical wear facet is present on the lingual surface of each tooth crown, and forms a considerable angle with the corresponding root. The maxillary teeth are much wider labiolingually than anteroposteriorly at the bases of their crowns, one of the middle maxillary tooth roots being about twice as wide labiolingually as anteroposteriorly. Distinctive grooves run along the anterior and posterior surfaces of each maxillary tooth root, and the anterior groove is deeper than the posterior one. Each maxillary tooth crown is considerably wider both anteroposteriorly and labiolingually than the corresponding root.

In contrast to the maxillary dentition, the dentary teeth are most extensively exposed in lingual view. Enamel is present on the lingual sides of the dentary tooth crowns, but absent on the labial sides. The prominent primary ridge is anteriorly offset on the lingual side of each dentary tooth crown, but the offset is less than in the maxillary teeth. The primary ridge is prominent throughout its length and extends onto the labial surface of the root. The anterior and posterior marginal ridges are weaker than in the maxillary teeth. Extremely weak secondary ridges are present posterior to the primary ridge on the lingual surface of the crown. Coarse denticles are seen along the edges of some dentary tooth crowns, extending for a short distance onto the lingual crown surface. The dentary tooth crowns each bear a prominence at the base of the labial surface, forming a buccal shelf as in other leptoceratopsids [2]. A large, nearly vertical wear facet is present on the labial side of each dentary tooth crown. The dentary tooth crowns are anteroposteriorly wider, but labiolingually narrower, than the corresponding roots. The crowns are deflected laterally from the roots at an angle of about 40 degrees. As in the maxillary teeth, the anterior and posterior surfaces of the dentary tooth roots bear grooves. In one anterior dentary tooth, the root is at least twice as long apicobasally as the crown.

Fourteen articulated presacral vertebrae are preserved, though the first (the atlas) and last (the fourth dorsal vertebra) of the series are represented only by a partial centrum and a partial arch, respectively (Figures 5 and 6). Based on the locations of the parapophyses, ten vertebrae of the preserved series are identified as cervicals and four as anterior dorsals. The centra, although not the arches, of the first three cervical vertebrae are partially fused together.

The axial centrum is probably the longest in the preserved series, although the centrum of the third cervical vertebra (Ce3) is only slightly shorter (Figures 5A and B). The centra of the subsequent cervical vertebrae are considerably shorter anteroposteriorly than those of the axis and Ce3, a feature also seen in ceratopsids but not in typical non-ceratopsid ceratopsians such as *Protoceratops* [25]. The fourth and more posterior cervical centra are very short anteroposteriorly (e.g., Ce4 has a centrum height/length ratio of about 2.0). The three preserved dorsal centra are proportionally even shorter, with centrum height/length ratios of more than 2.0. The preserved presacral centra are amphicoelous, the anterior ones having particularly concave inter-central articular surfaces.

All preserved centra are ventrally keeled, but the keels vary in size. The ventral keel of the axial centrum is transversely wide and extremely weak. Sharp and prominent ventral keels are present on the centra of Ce3 and Ce4, but the keels of the more posterior cervicals become lower and wider. The three anteriormost dorsal vertebrae (D1–D3) have much sharper ventral keels than any of the cervical vertebrae. The keel on the centrum of D2 is especially prominent, being both transversely narrower and dorsoventrally higher than those of the other preserved vertebrae. The lateral surfaces of the preserved cervical centra each bear two large fossae divided by a horizontal thick ridge emanating from the parapophysis. This feature is variably developed in many neoceratopsians, including ceratopsids [24], [25], [27]. In *Zhuchengceratops* the horizontal ridge is very subdued on the axial centrum, and its prominence increases in more posterior vertebrae until it reaches a maximum in Ce5 and Ce6. In the most posterior cervical vertebrae, by contrast, this ridge is barely present. In Ce5 through Ce7, the dorsal fossa is itself subdivided by another horizontal ridge, and the ventral fossa is similarly subdivided in the seventh through tenth vertebrae. Dorsal and ventral fossae, divided by a horizontal ridge, are also present on the lateral surfaces of the three anteriormost dorsal centra. A distinct, anteroposteriorly elongate nutrient foramen is located in the center of the ventral fossa in Ce2 through Ce8. Similar foramina are also seen in the cervical vertebrae of some ceratopsids, such as *Triceratops* [27]. In lateral view, the anterior and posterior edges of all preserved vertebral centra are prominent, and the inter-central articular surfaces of the anterior and middle cervical vertebrae are considerably everted.

The laminar system is well developed in the preserved vertebrae, particularly in the posterior cervicals and anterior dorsals. In the axis and Ce3, the parapophysis extends posterodorsally as a short ridge, though in the latter vertebra the ridge is comparatively weak. As described above, three different horizontal ridges are variably present on the lateral surface of the centrum in the preserved vertebrae. Anterior and posterior centrodiapophyseal laminae are present in the axis through Ce9, and are most prominent in the middle cervicals. They are also prominent in the posterior cervical vertebrae, but approach one another so closely that they are nearly fused. A prezygodiapophyseal lamina is present in all preserved vertebrae, and becomes more prominent posteriorly along the series. A postzygodiapophyseal lamina is present in the 7^th^ through 13^th^ presacral vertebrae, though in the 7^th^ vertebra the lamina is extremely small. In Ce3, the diapophysis sends posterodorsally a short ridge, possibly homologous to the postzygodiapophyseal laminae seen in some of the other vertebrae. A paradiapophyseal lamina is present in the 10^th^ through 13^th^ presacral vertebrae, while anterior and posterior centroparapophyseal laminae are present in D1 through D3. Centroprezygapophyseal and centropostzygapophyseal laminae are present in all preserved vertebrae. A prezygopostzygapophyseal lamina is present in Ce3 through Ce8. Very weak prespinal and postspinal laminae are present in at least the anterior cervical vertebrae.

The axial parapophysis is located at about the mid-height of the lateral surface of the centrum. The parapophysis migrates posterodorsally in the more posterior cervicals, and the parapophysis of the last cervical merges with the ventral surface of the diapophysis on the suture between the centrum and the neural arch. The diapophysis of the axis is located slightly posterior to the mid-length of the vertebra, and dorsal to the suture between the neural arch and centrum. The diapophysis is more anteriorly located in the remainder of the series, and moves dorsally in the more posterior vertebrae. The base of the diapophysis remains considerably ventral to the level of the zygapophyses in the anterior and middle cervical vertebrae, but lies at about the level of the zygapophyses in the posterior cervical and anterior dorsal vertebrae. In the anterior and middle cervical vertebrae, the diapophysis is nearly horizontally oriented and somewhat rod-like. In the seventh and more posterior cervical vertebrae the diapophysis is more dorsally oriented, and this is particularly true in the posteriormost cervicals. However, the diapophyseal facet always faces laterally rather than dorsolaterally. The diapophyses of the posterior cervical vertebrae are much larger, with respect to both robustness and length, than those of the anterior and middle ones. The posterior cervical diapophyses also differ from their anterior counterparts in being somewhat strap-like, due to the significant development of the prezygodiapophyseal lamina in the posterior cervicals. The diapophyses of the posteriormost cervical vertebrae are T-shaped in cross section, since the postzygodiapophyseal and paradiapophyseal laminae are also prominently developed.

The pedicles of each vertebral arch are high relative to the corresponding centrum, roughly equaling the height of the centrum at the posterior end. The pedicles are proportionally higher in *Protoceratops* [25] but lower in ceratopsids [27]. Pedicle height decreases posteriorly along the vertebral column of *Zhuchengceratops*. The postaxial cervical neural arches appear only slightly constricted in lateral view, due to the prominence of the centropostzygapophyseal laminae. In the posterior cervical and anterior dorsal vertebrae the centroprezygapophyseal laminae are also prominent, so that the anterior edges of the centra and arches are approximately straight in lateral view. The nearly vertically oriented zygapophyses are enlarged, so that the pre- and postzygapophyses are only separated by a small gap in lateral view.

The cervical neural spines are variable in size, location, orientation and shape. Most of the axial neural spine is missing, but the preserved posterior margin suggests that the intact spine was nearly vertical. In general, however, the anterior neural spines are slightly anteriorly inclined, the middle ones are nearly vertical, and the posterior ones are posteriorly inclined. The posterior neural spines are more posteriorly oriented (about 45 degrees to the vertical) than in other non-ceratopsid ceratopsians. Most of the neural spines are plate-like, but that of Ce9 is somewhat rod-like because the spinoprezygapophyseal and spinopostzygapophyseal laminae are only weakly developed. The neural spines are much greater in height than in anteroposterior breadth. At least in Ce3, the posterior margin of the plate-like neural spine is considerably thicker transversely than the anterior margin.

The cervical ribs are represented by seven relatively complete examples (Figure 7) and a few fragments. The atlantal rib is a single-headed, somewhat spoon-shaped element (Figures 7A and B). It is more than twice as long as the axial centrum. The proximal part of the rib shaft is strongly expanded dorsoventrally to form a plate-like structure that is somewhat rounded in lateral view, with a strongly concave medial surface and a convex lateral surface. The articular surface for the atlas is flat and semilunate in outline. The rib shaft is slender and strongly flattened mediolaterally, though its distalmost portion is thickened along the ventral margin. The axial rib is considerably longer than the atlantal rib, and in fact is probably the longest of all the cervical ribs. The proximal end is double-headed, but relatively unexpanded, with the tuberculum and capitulum close together (Figures 7C and D). The tuberculum is mediolaterally thin and is oriented dorsomedially, extending nearly perpendicular to the long axis of the rib shaft. The capitulum is much more robust, terminating in a nearly circular articular facet. The rib shaft is long and slender, with a markedly convex lateral surface. The third cervical rib is in general similar to the axial rib, but differs in a few features: in the third rib the shaft is much more robust, the tuberculum and capitulum are widely separated from each other, the tuberculum is less dorsally oriented, the area where the tuberculum and capitulum come together bears a longitudinally oriented crest along the lateral surface and a groove along the medial surface, and a small accessory process occurs at the mid-length of the dorsal margin of the rib shaft (Figures 7E and F).

The fourth and subsequent cervical ribs differ from the third cervical rib in that the tuberculum and capitulum are more rod-like and strap-like, respectively. In these posterior cervical ribs the tuberculum is nearly aligned with the rib shaft (Figures 7G and H), whereas the capitulum diverges at a large angle from the long axis of the rib shaft (the reverse is true for the axial and third cervical ribs). The fifth cervical rib has a prominent accessory dorsal process (Figures 7I and J), and in this rib the lateral crest at the confluence of the capitulum and tuberculum is unusually large. Distal to the accessory dorsal process the rib shaft becomes somewhat rod-like, although the distalmost portion of the rib shaft deviates from this shape by widening dorsoventrally. The sixth cervical rib has a shaft much wider than those of the preceding cervical ribs, and the accessory dorsal process is so enlarged that the distal part of the rib shaft is essentially bifurcated (Figures 7K and L). A shallow, longitudinal groove is present on the lateral surface of the ventral process of the rib shaft. A more posterior cervical rib has an even wider shaft, but in the case of this rib the lateral crest is absent, the tuberculum has little medial curvature, and the shaft is not bifurcated although the accessory dorsal process is relatively large (Figures 7M and N). A lateral crest and an accessory dorsal process are also seen in the middle and posterior cervical ribs of some ceratopsids, such as *Triceratops* [27]. None of the cervical ribs figured by Brown and Schlaikjer for *Montanoceratops* shows a well-developed dorsal process [28].

Most of the right dorsal ribs are preserved (Figures 1 and 8). They are considerably curved medially, though the curvature is less pronounced in the first two dorsal ribs and the posteriormost dorsal ribs are nearly straight. The dorsal ribs lengthen posteriorly until the fifth in the series, which appears to be the longest rib of *Zhuchengceratops*. Moving posteriorly from this point, the dorsal ribs become shorter in length. There is a continuous narrowing of the dorsal ribs posteriorly along the series, with shaft width decreasing by about two-thirds from the anteriormost dorsal rib to the posteriormost. The tuberculum is prominent on the most anterior dorsal ribs, becomes smaller posteriorly along the series, and is barely present beyond the sixth dorsal rib. A longitudinal groove runs along the posterior surface of the proximal third of each anterior and middle dorsal rib, but is probably absent in the posterior dorsal ribs. The shafts of the posterior dorsal ribs are more rod-like than strap-like.

## Discussion

In our numerical phylogenetic analyses, *Zhuchengceratops* is recovered as a derived leptoceratopsid (Figure 9). It is posited in an unresolved position within a clade composed of *Zhuchengceratops*, *Udanoceratops*, and *Leptoceratops* (Figures 9A and B) based on the matrices of both Makovicky (2010) and Chinnery and Horner (2007).

The leptoceratopsid affinities of *Zhuchengceratops* are indicated by the presence of many derived features seen in other leptoceratopsids [14], [29], [30]: dentary teeth with horizontal, labially positioned shelves, dentary teeth bulbously expanded at root-crown transition on labial side, primary ridge inset relative to cingulum on each maxillary tooth, mandible deep, dorsal margin of dentary strongly concave in lateral view, anterior part of dorsal margin of dentary considerably dorsal to tooth row, posterior edge of coronoid process of dentary notched, surangular without distinct lateral ridge overhanging angular, and dorsal vertebrae with flat zygapophyseal facets. Furthermore, *Zhuchengceratops* shares two derived features with both *Leptoceratops* and *Udanoceratops*: ventral margin of maxilla convex in lateral view and jaw articulation above tips of dentary teeth.

Our numerical analyses could not fully resolve the systematic position of *Zhuchengceratops* (Figure 9). The available morphological data are in fact contradictory regarding the relationships of *Zhuchengceratops*, *Udanoceratops*, and *Leptoceratops*. *Zhuchengceratops* has a dorsoventrally very deep maxilla, a derived feature probably also present in *Udanoceratops* [1], [8] but absent in *Leptoceratops*. However, *Zhuchengceratops* also possesses several derived features seen in *Leptoceratops* [18] but unknown in other ceratopsians: upward curvature of ventral margin restricted to anteriormost part of mandible, posterior end of mandible with sharp ventral deflection, contact between dentary and post-dentary bones nearly vertical (also seen in ceratopsids), and hyoid bones rod-shaped. Accordingly, the available morphological data support, albeit equivocally, the interpretation that *Zhuchengceratops* was more closely related to *Leptoceratops* than to *Udanoceratops* (it should be noted that not all of these features were included in the numerical phylogenetic analyses).

The Ceratopsidae was until recently the only known species-rich clade among the Ceratopsia, and unquestionably represents one of the last radiations of the ornithischian dinosaurs [4], [24]. Another speciose clade, the Bagaceratopsidae, is recognized by some authors [31], [32]. Besides *Protoceratops* and *Bagaceratops*, other nominal taxa that have been suggested to belong to this group include *Gobiceratops minutus* [33], *Breviceratops kozlowskii* [34], [35], *Lamaceratops tereschenkoi* [36], *Platyceratops tatarinovi* [36], and *Magnirostris dodsoni* [31]. *Ajkaceratops kozmai* may also be closely related to the bagaceratopsids [32]. However, most of these taxa have had their validity questioned [22], [32], so that the number of legitimate species that can be assigned to the Bagaceratopsidae is presently uncertain. By contrast, a recent phylogenetic analysis places eight well-established species in the Leptoceratopsidae, including the Asian taxa *Udanoceratops* and *Asiaceratops* [4], [6]–[8]. *Zhuchengceratops* is the third leptoceratopsid reported from Asia. Consequently, the Leptoceratopsidae represents the second strongly supported speciose ceratopsian clade, indicating that an unusual, diverse group of apparently less specialized ceratopsian dinosaurs coexisted with the Ceratopsidae during the Late Cretaceous [4].

Within the context of the leptoceratopsid phylogeny presented in Figure 9, the Leptoceratopsidae are inferred to have originated in Asia. However, one interesting implication of the phylogeny is that dispersals took place in both directions across the Bering Strait in the Late Cretaceous: at least once from Asia to North America and once in the opposite direction. This is significant given that a predominant direction of dispersal from Asia to North America has been suggested for many other vertebrate groups [37], [38].

Interestingly, a basal centrosaurine ceratopsian has been recently reported from Wangshi Group strata exposed at the Zangjiazhuang locality [38], 5 km away from the Kugou site that produced the holotype of *Zhuchengceratops inexpectus*. The discovery of a leptoceratopsid from the Wangshi Group of Zhucheng thus indicates that leptoceratopsids and ceratopsids coexisted in the Late Cretaceous of Shandong, China. This is significant because the two groups had previously been known to occur together only in the uppermost Cretaceous deposits of North America [10].

*Zhuchengceratops* is significantly different from other members of the group. It displays several features previously unreported in leptoceratopsids but seen in more derived ceratopsians. As in ceratopsids, the dorsal surface of the posterior process of the maxilla contacts the pterygoid and ectopterygoid, while the posterodorsal margin of the maxilla has a restricted articulation with the jugal [24]. The posterior end of the jugal expands dorsally to form the posteroventral border of the infratemporal fenestra, a morphology similar to that seen in centrosaurines and some chasmosaurines [24]. The pterygoid of *Zhuchengceratops* resembles those of ceratopsids and *Protoceratops* [22] in bearing a ventral ridge that defines a ‘Eustachian canal’, and in that the quadrate ramus forms a large angle with the palatine process. The distal end of the coronoid process is slightly expanded anteroposteriorly, resembling to some extent the ceratopsid condition [24]. The posterior end of the dentary tooth row is only slightly anterior to the posterior margin of the coronoid process, a feature more reminiscent of ceratopsids than of other non-ceratopsid ceratopsians. As in ceratopsids, the nearly vertically oriented contact between the dentary and post-dentary bones lies posterior to the coronoid process. The prearticular of *Zhuchengceratops* contacts the dentary, a derived feature also seen in *Protoceratops, Bagaceratops*, and ceratopsids [2]. The axial and third cervical centra are longer than those of the more posterior cervicals, a feature also seen in ceratopsids [25]. As in *Triceratops* and some other ceratopsids [28], large nutrient foramina are present on the lateral surfaces of the cervical centra, and the middle cervical ribs are very robust and bear prominent accessory dorsal processes.

These features are consistent with the phylogenetic hypothesis that the leptoceratopsids are closer to the Ceratopsidae than are the Protoceratopsidae [10]. However, our analysis supports the alternative hypothesis that the Protoceratopsidae are more closely related to the Ceratopsidae [2]. In the framework of the phylogenetic hypothesis presented in this paper, the above-mentioned similarities between *Zhuchengceratops* on the one hand, and ceratopsids and their close relatives on the other, are interpreted as convergences. Consequently, *Zhuchengceratops* seems to have retained a basically leptoceratopsid feeding system while independently evolving some ceratopsid-like osteological features. These features increase the known morphological disparity within the Leptoceratopsidae. It should be noted, however, that the recovered inter-relationships among the Coronosauria are poorly supported by the available data as indicated by the relatively low Bremer Support and Bootstrap values (Figure 9). A more comprehensive dataset will eventually be required in order to better understand coronosaurian phylogeny, given the current controversy over the systematic position of the Leptoceratopsidae [2], [7], [10], [14].

The discovery of *Zhuchengceratops* provides new evidence for the hypothesis that leptoceratopsids represent a successful radiation, paralleling that of ceratopsids, in the Late Cretaceous of Asia and North America [4], [6], [8], [38]. Interestingly, these two species-rich clades both possess many modifications related to mastication, but they modified their jaws and dentition in different ways. The leptoceratopsids have a short parietosquamosal frill, a massive lower jaw, a dorsally located mandibular glenoid articulation, and relatively massive teeth with a few distinctive features (e.g., prominences on the labial surfaces of the dentary crowns, and a vertical-notch tooth wear pattern). The ceratopsids have a much longer parietosquamosal frill, a shallow lower jaw, a ventrally located mandibular glenoid articulation, and highly specialized slender teeth (e.g., more than two replacement teeth in each alveolus, and teeth with two roots). These two different sets of highly specialized jaw and dental features may reflect adaptation to different food resources, but their occurrence in two closely-related lineages that coexisted and radiated in the same broad geographic areas during the Late Cretaceous is nevertheless surprising.
